# Healing potentials of polymethylmethacrylate bone cement combined with platelet gel in the critical-sized radial bone defect of rats

**DOI:** 10.1371/journal.pone.0194751

**Published:** 2018-04-02

**Authors:** Ahmad Oryan, Soodeh Alidadi, Amin Bigham-Sadegh, Ali Moshiri

**Affiliations:** 1 Department of Pathology, School of Veterinary Medicine, Shiraz University, Shiraz, Iran; 2 Department of Pathology, School of Veterinary Medicine, Shiraz University, Shiraz, Iran; 3 Department of clinical Sciences, School of Veterinary Medicine, Shahrekord University, Shahrekord, Iran; 4 Department of Orthopedic Surgery, Faculty of Medicine, AJA University of Medical Science, Tehran, Iran; Kyoto Daigaku, JAPAN

## Abstract

Polymethylmethacrylate (PMMA) is the most commonly used filler material that lacks biological properties and osteoconductivity or osteoinductivity. Platelet gel (PG) is a typical source of growth factors, cytokines and molecules efficient for bone formation and remodeling. The aim of this study was to evaluate bone healing and regeneration of bone defect in rat model by combining PMMA with PG. A total of 50 defects were created in the diaphysis of the radii of 25 male Sprague-Dawley rats. These defects were randomly divided into five groups (n = 10 defects for each group) and treated by autograft, plain PMMA, PG and PMMA-PG or left untreated. The rats were examined clinically and radiologically during the experiment and also after euthanasia at the 8^th^ post-operative week, the healed defects were evaluated by gross morphology, histopathology, histomorphometry, computed tomography, scanning electron microscopy and biomechanical testing. PG could function as efficiently as autograft in promoting bone healing of the radial bones. Additionally, bone formation, and densities of cartilaginous and osseous tissues in the defects treated with autograft, PG and PMMA-PG were more satisfactory than the untreated and PMMA treated defects. Compared with the PMMA-PG implant, more PMMA residuals remained in the defect area and induced more intense inflammatory reaction. In conclusion, addition of PG could improve the bone regenerative properties of PMMA bone cement compared with PMMA alone *in vivo*. Therefore, the PG-PMMA can be proposed as a promising option to increase regenerative potential of PMMA, particularly when it is used as fixator, filler or adhesive in the dentistry, neurosurgery and bone tissue engineering applications.

## Introduction

Treatment of large bone defects remains as a major challenge in the field of orthopedics and regenerative medicine. Although implantation of the autologous bone grafts is still considered as the gold standard, some drawbacks such as limited availability and donor site morbidity have limited the use of these grafts [1]. Bone tissue engineering (BTE) has suggested many options for promoting bone healing and regeneration based on the use of growth factors and cells along with the scaffolds. Nonetheless, the efforts to find a more appropriate and optimal approach continue. Among many potential bone cement materials, acrylic bone cement or polymethylmethacrylate (PMMA) has been used in the orthopedic procedures [2, 3]. This polymer has been proposed to be suitable in fixation of prosthetic implants and repair of vertebral fractures or vertebroplasty [4–7]. Other applications of PMMA in the orthopedic field include arthroplasty, remodeling of osteoporotic bones, hip endoprosthesis, hip replacement, and cranioplasty [4, 5]. PMMA can also conform to the shape of its surrounding tissue, form mechanical bonds with implants and provide mechanical support [2].

Despite of these beneficial characteristics, PMMA is associated with some disadvantages such as poor osteointegration and minimal bone attachment effect that should be augmented biologically [4, 8]. In addition, this self-curing acrylic polymer has no adhesive properties to bone surfaces and no bioactivity [9]. Many studies have taken consideration into both mechanical and biological properties, osteoindctivity, and osteoconductivity and bioactivity of PMMA by addition of bioactive materials [10–12]. In this regard, bioceramics such as hydroxyapatite (HA), tricalcium phosphate (TCP) or phosphate glasses composites have been used in many studies [9, 10, 13]. For example, Kim et al. fabricated a new bioactive bone cement consisting PMMA bone cement, HA and chitosan powder and could enhance cell attachment and proliferation, bioactivity and osteoconductivity of pure PMMA *in vitro* and *in vivo* [2]. Additionally, Aghyarian and coworkers demonstrated that composite PMMA-HA/brushite bone cement increased the healing potentials of PMMA for spinal augmentation by providing high mechanical strength and essential bioactivity [14]. Nevertheless, continuous attempts to modify the biological and regenerative characteristics of PMMA to enhance its properties are in progress.

Platelet-rich plasma (PRP) which is biodegradable, biocompatible and bioactive and has osteoinductive and osteoconductive capacities can be considered as an appropriate candidate to be added into PMMA [15, 16]. It contains different types of concentrated growth factors which exert beneficial effects on bone regeneration [17–19]. The autologous PRP has particularly been widely investigated and demonstrated its healing efficacy in many studies [20–22]. Marx et al. [23] applied PRP as a source of autogenous growth factors in regeneration of maxillofacial defects in humans and found that PRP promoted maturation of autogenous bone grafts with a higher bone density. PRP can be used in different forms such as injective liquid, gel, sponge and hydrogels [19, 24]. It can be activated to form a gel by addition of calcium chloride or thrombin alone or in combination [25]. This platelet gel (PG) may be applied alone or in combination with different components to provide bone regenerative substitutes [18, 25]. However, the effectiveness of PRP is debated and some studies could not achieve positive and promising outcomes [20, 21, 25]. Hence, considering wide application of PMMA, we proposed a new approach to fabricate a bioactive PMMA-based composite by adding PG to the commercial PMMA bone cement and evaluated the healing potential of PMMA with or without xenogenous PG in a rat critical-sized radial defect model.

## Materials and methods

### Preparation of materials

Xenogenous human derived PRP was provided from the Shiraz Blood Bank Center. The PRP was firstly maintained at -20°C for 24 h, then freeze-dried at -80°C and a pressure of 1 mBar for 48 h (freeze dryer ALPHA 2–4 LD plus Martin Christ, Germany) and transformed to powder. The powder was sterilized by ^60^Co γ-irradiation at a dose of 15 kGy for 10 min and was dissolved in sterile phosphate buffered saline (PBS, 0.9% NaCl) [26]. Five ml of PRP was activated by adding 5000 U bovine thrombin and five ml of 10% CaCl2 with a proportion of 10 platelet solution to 1 activator to produce PG [26–28]. Number of platelets in the whole blood and PG was 259.4 ± 41.6 × 10^3^/μl and 1174.3 ± 261.3 ×10^3^/μl (4.5-fold higher than platelets of the whole blood), respectively. Moreover, the health and activity of platelets were examined and confirmed by the Shiraz Blood Bank Center.

The two-component PMMA bone cement used in this experiment was obtained from Osteopal® (Biomet, Merck, Germany). The pure PMMA samples were prepared by adding the methylmethacrylate monomer (MMA) to the pre-polymerized PMMA powder at a ratio of 1.52 g/ml [2, 10]. The mixture was stirred until formation a smooth paste. The resultant dough was divided into several pieces (2 × 2 × 5 mm^3^) before the polymerization process was completed. After the curing time, the prepared PMMA samples were washed with distilled water and sterilized under ^60^Co γ-irradiation at the dose of 15 kGy (λ = 254 nm) for 10 min and kept in sterile packs until use.

The PMMA-PG composites were prepared using the following method. The sterilized PRP powder was mixed with sterile PBS to obtain a dispersion with a 20% v/v concentration. It was then mixed with an equal proportion of the previously prepared mixture of MMA and PMMA for 40 s and homogenized. The resulting dough was converted to a paste and then it was cut into several pieces (2 × 2 × 5 mm^3^). As the PRP was absorbed by the PMMA cement, the PMMA-PRP composite was suspended in a solution containing PRP activator composed of a mixture of 5000 U bovine thrombin per five ml of 10% CaCl_2_. Consequently, the PG was formed inside the PMMA cement [1, 27]. The implants were then kept at -20°C for 24 h, then freeze-dried at -80°C for 48 h. Finally, they were sterilized under ^60^Co γ-irradiation at the dose of 15 kGy for 10 min and kept in sterile packs until further use. In addition, the ultra-structure of PMMA and PMMA-PG were examined by scanning electron microscopy (SEM, Cambridge, London, UK)).

### Ethics

Human care for all animals was provided in accordance with the Guide for Care and Use of Laboratory Animals published by the National Institutes of Health (NIH publication No. 85–23, revised 1985). The present study was approved by the local Ethics Committee of “Regulations for using animals in scientific procedures” in School of Veterinary Medicine, Shiraz University, Shiraz, Iran.

### Animals and surgical procedure

Twenty-five mature male Spraue-Dawley rats weighing 250 ± 25 g were purchased from the Razi Institute, Karaj, Iran. The animals had full access to standard food and water ad libitum throughout the study. General anesthesia was administered by intramuscular injection of Ketamine hydrochloride (Ketamine 10%, 50 mg/kg), Xylazine (Xylazine 2%, 2 mg/kg), and Acepromazine maleate (1mg/kg; all from Alfasa Co., Woerden, Holland). Under aseptic condition, a 2-cm incision was bilaterally made over the forearm and the radii of each animal were exposed. Using an electrical bone saw (Strong. Co. Seoul, South Korea), five mm of the diaphysis of each radius was cut under saline dripping. The created bone defects (n = 50, 10 defects in each group) were either treated with autograft, PMMA, PG alone, PMMA-PG or left empty (defect or untreated group). It should be mentioned that the bone segments harvested from the radii in the defect/untreated group were used as the autologous bone grafts for the autograft group in the same rat. After inserting the implants in the defect areas, the muscles, subcutaneous fascia and skin were sutured in a routine fashion. Post-operative analgesia and antibiotic therapy were provided by intramuscular administration of flunixin meglumine (Razak Co., Tehran, Iran; 2.5 mg/kg) and enrofloxacin (Enrofan 5%, Erfan, Tehran, Iran), respectively for 5 days.

### Clinical examination

The animals were examined in terms of their clinical behavior, physical activity and weight bearing of the injured forelimbs after surgery. In addition, the clinical status of the injured area such as post-surgical swelling, hyperemia, pain and other clinical signs were checked on digital palpation.

### Radiological evaluation

To evaluate bone formation and the healing degree of the defects, plain lateral radiographs of the forelimbs were taken at the 2^nd^, 5^th^, and 8^th^ weeks post injury. The results were scored according to the modified Lane and Sandhu scoring system [29] (Table 1).

**Table 1:** Modified Lane and Sandhu radiological scoring system.

Bone formation	
No evidence of bone formation	0
Bone formation occupying 25% of the defect	1
Bone formation occupying 50% of the defect	2
Bone formation occupying 75% of the defect	3
Bone formation occupying 100% of the defect	4
Union (proximal and distal ends were evaluated separately)	
No union	0
Possible union	1
Radiographic union	2
Remodeling	
No evidence of remodeling	0
Remodeling of medullary canal	1
Full remodeling of cortex	2
Total points possible per category	
Bone formation	4
Proximal union	2
Distal union	2
Remodeling	2
Maximum score	10

### Gross evaluation

After eight weeks of bone injury, the animals were euthanized and their forelimbs were removed [1]. The radii were evaluated in terms of the degree of healing and the tissue that filled the defect sites at the macroscopic level and then they were blindly scored as follows: complete union and the presence of bridging bone (+3 score), incomplete union with the presence of cartilage (+2) or soft tissue (+1) within the defect, and no union or instability at the defect site (0 score) [30].

### Three-dimensional computed tomography (3D-CT)

The bone specimens were examined by CT-scan, using Inveon TM unit (Siemens Healthcare USA, Inc., PA, USA). They were scanned at 0.06-mm thickness sections at the longitudinal and transverse views. The images were reconstructed by Inveon Research Workplace software (Siemens Healthcare USA, Inc., PA, USA) to create 3-D images of the newly formed bone. Bone volume of the bone defects reported as percentage was calculated from the acquired images by the software ImageJ (version 1.51 Mac, National Institutes of Health, USA; http://imagej.nih.gov/ij), based on the following formulation and analyzed statistically.

$$\%Bone\;volume=\frac{volumeofthedefectoccupiedbynewbone}{volumeoftheinitialdefect}\times 100$$

### Histopathologic and histomorphometric evaluations

The bone samples (n = 5 for each group) were fixed in 10% neutral buffered formalin and then decalcified with 15% formic acid. The specimens were then dehydrated in graded ethanol solution and embedded in paraffin wax. The samples were sectioned at 5-μm thickness and then stained with hematoxylin and eosin. The bone specimens were examined by a light microscope (Olympus, Tokyo, Japan) connected to a digital camera (Olympus DP71) and blindly scored based on the tissue type that filled the defect sites including bone, hyaline cartilage and fibrous connective tissues [30]. In addition, number of fibroblasts + fibrocytes, chondroblasts + chondrocytes, osteoblasts + osteocytes, osteoclasts, osteons, inflammatory cells including neutrophils, lymphocytes, plasma cells, macrophages and giant cells, and other constituents including blood vessels were blindly counted and analyzed for histomorphometric evaluation. Moreover, the densities of fibrous connective tissue (FCT), cartilaginous (CT) and osseous tissue (OT) (%) were calculated and analyzed. For cell counting, the photomicrographs were captured from the histological fields and then transferred to the computer software (Adobe Photoshop CC, extended version; CA, USA) for digital analysis performed blindly by two expert healing pathologists. In each group, 10 histopathologic fields (× 100) from two bone sections and thus, in total 100 microscopic fields were evaluated and counted [1, 31].

### Scanning electron microscopy

The bone samples were fixed in cold 2.5% glutaraldehyde, dehydrated in graded series of ethanol and finally gold coated. High-qualified images with different KVs and magnifications were created by a SEM. The quality and degree of healing were evaluated and compared amongst different groups. Different structures including collagen fibers, degree of calcification of the matrices, Haversian canals, and osteons were evaluated.

### Biomechanical evaluation

The radius- ulna complexes (n = 5 for each group) were wrapped in PBS-soaked gauzes and frozen at -20°C until testing. Biomechanical test was conducted on the specimens, using a universal tensile testing machine (Instron, London, UK) [1, 15, 28, 30, 32]. To perform the three-point bending test, the bone samples were placed horizontally on two supporting bars at a distance of 16 mm. The third bar was lowered at the middle of the diaphysis where the defect site placed. The force was loaded at a rate of 5 mm/min on the bones until fracturing occurred. The load-deformation curves were sketched by the machine and the data obtained from the curves including maximum load, strain, stress, and stiffness were calculated, expressed as Mean ± standard deviation (SD) and analyzed in each group.

### Statistical analysis

The data achieved from the histomorphometric examination were expressed as mean ± SD and analyzed by one-way ANOVA with subsequent Tukey post-hoc tests. The scored values and biomechanical data were statistically compared by Kruskal-Wallis H, non-parametric ANOVA test, and when they were significant, by Mann-Whitney U test. A p-value less than 0.05 was considered to be statistically significant. Statistical analyses were performed by SPSS software, version 16.0 (SPSS, Inc, Chicago, USA).

## Results

### Morphology of scaffolds

**Fig 1:**
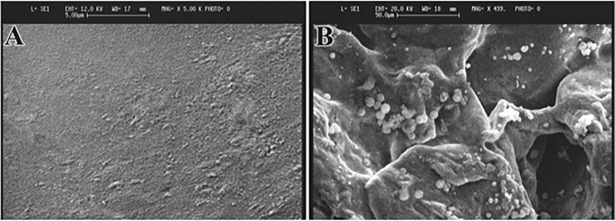
Scanning electron micrographs of the pure PMMA bone cement and PMMA-PG scaffold.

The SEM micrographs of the PMMA bone cement and PMMA-PG scaffold showed that the pure PMMA was less rough and porous than the PMMA-PG scaffold, so that platelets were observed in some parts of the scaffold (Fig 1).

### Clinical manifestations

All the animals had good appetite and physical activity during the experiment duration and there was no death. Edema, hyperemia, swelling and pain were detectable at the surgery region, in all groups at the first two weeks after the operation. These signs rapidly reduced toward the normal status in the defects treated with PG, PMMA-PG and autograft, so that they appeared almost normal at the 3^rd^ post-operative week. After eight weeks, the untreated defects were empty under digital palpation. In the PG and autograft groups, the defects were filled with a new firm tissue so that the defect site was not detectable. The defects in the PMMA-PG group were filled with a new tissue which was not as firm as the tissue in the PG and autograft groups. The defects treated with PMMA seemed to be filled with a new soft tissue. Regardless of the above descriptions, all animals in the experiment had weight bearing because of the supportive role of the ulna.

### Gross morphology

**Fig 2:**
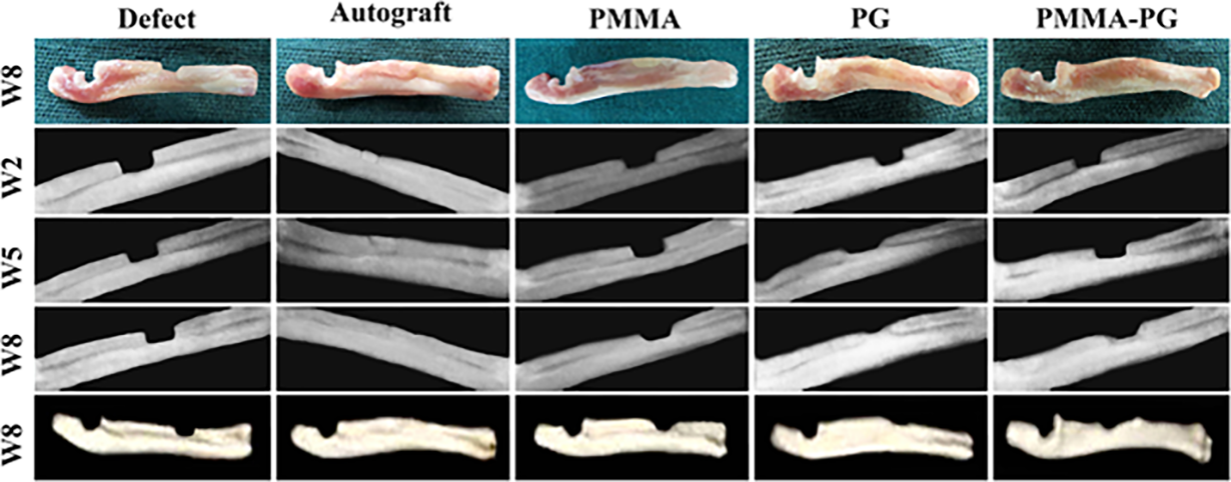
Macroscopic and diagnostic imaging findings of the critical sized segmental radial defect model in rats. Eight weeks after injury, the defect group was filled with fibrous tissue or remained empty, while the autograft group was replaced by firm tissue. The PMMA bone cement was not degraded and mostly replaced by soft tissue. PG was completely degraded and filled with firm cartilage or bone. Very small remnant of the PMMA-PG implant was visible and the soft or firm tissue filled the defects in this group. The defects in PG and autograft groups showed greater macroscopic scores compared with those in the defect and PMMA groups (*P<0*.*05*). The autograft group had significantly higher radiographic scores than other groups after two and five weeks (*P<0*.*05*). The PG group had greater radiographic scores than the defect group at the 5^th^ week post-operation (*P = 0*.*034)*. At the 8^th^ week, the autograft had higher radiographic scores than other groups with the exception of the PG group and the PG and PMMA-PG groups were superior to the PMMA and defect groups (*P<0*.*05*). The PG, PMMA-PG and autograft groups had significantly higher bone volume than the defect and PMMA groups (*P<0*.*05*) after eight weeks of injury.

After eight weeks, the untreated defects were replaced with a soft tissue similar to fascia and the defects were almost empty (Fig 2). The defects treated with PG and autograft were filled with firm tissues and bone union seemed almost complete. In the PMMA group, the implant was not still completely degraded and the defect sites were mostly replaced by fibrous tissue along with the cement remnants. Eventually, firm tissues possibly cartilage or bone filled the defect sites in the PMMA-PG group, but bone union was still incomplete. In addition, very small segments of the PMMA-PG implant were still visible in the defect sites (Fig 2). The bone defects of the autograft and PG groups had significantly higher macroscopic scores compared with those of the defect and PMMA groups (*P<0*.*05*) (Table 2). The macroscopic scores related to the defects treated with PMMA-PG were significantly greater than those of the untreated defects (*P = 0*.*031*). Incorporation of PG into PMMA did not lead to significant difference in macroscopic scores between the PMMA and PMMA-PG groups (*P = 0*.*249*).

**Table 2:** Findings obtained from bone measurements at macroscopic and microscopic levels.

Type evaluation	Defect (1)	Autograft (2)	PMMA (3)	PG (4)	PMMA-PG (5)							
	Median (min-max)	Median (min-max)	Median (min-max)	Median (min-max)	Median (min-max)	P^a^	1 vs. 3	1 vs. 4	1 vs. 5	3 vs. 4	3 vs. 5	4 vs. 5
Macroscopic union *	1 (0–1)	3 (2–3)^b^	1 (1–2)	2 (2–3)	2(1–2)	*0*.*002*	*0*.*183*	*0*.*009*	*0*.*031*	*0*.*043*	*0*.*249*	*0*.*093*
Microscopic evaluation **	1 (1–2)	5 (4–6)^c^	2 (1–3)	5 (3–6)	3 (2–5)	*0*.*001*	*0*.*691*	*0*.*008*	*0*.*011*	*0*.*017*	*0*.*049*	*0*.*502*

PG: Platelet gel; PMMA: Polymethylmethacrylate

* Complete union (+ 3 score), presence of cartilage (+ 2 score), presence of soft tissue (+ 1 score), nonunion (0 score)

** Empty (0 score), fibrous tissue only (+ 1 score), more fibrous tissue than cartilage (+ 2 score), more cartilage than fibrous tissue (+ 3 score), cartilage only (+ 4 score), more cartilage than bone (+ 5 score), more bone than cartilage (+ 6 score) and bone only (+ 7 score)

^a^ Kruskal-Wallis non-parametric ANOVA

^b^ P = *0*.*008* and *0*.*020* (2 vs. 1 and 3)

^c^ *P = 0*.*005* and *0*.*011* (2 vs. 1 and 3) by Mann-Whitney U test

### Diagnostic imaging findings

**Fig 3:**
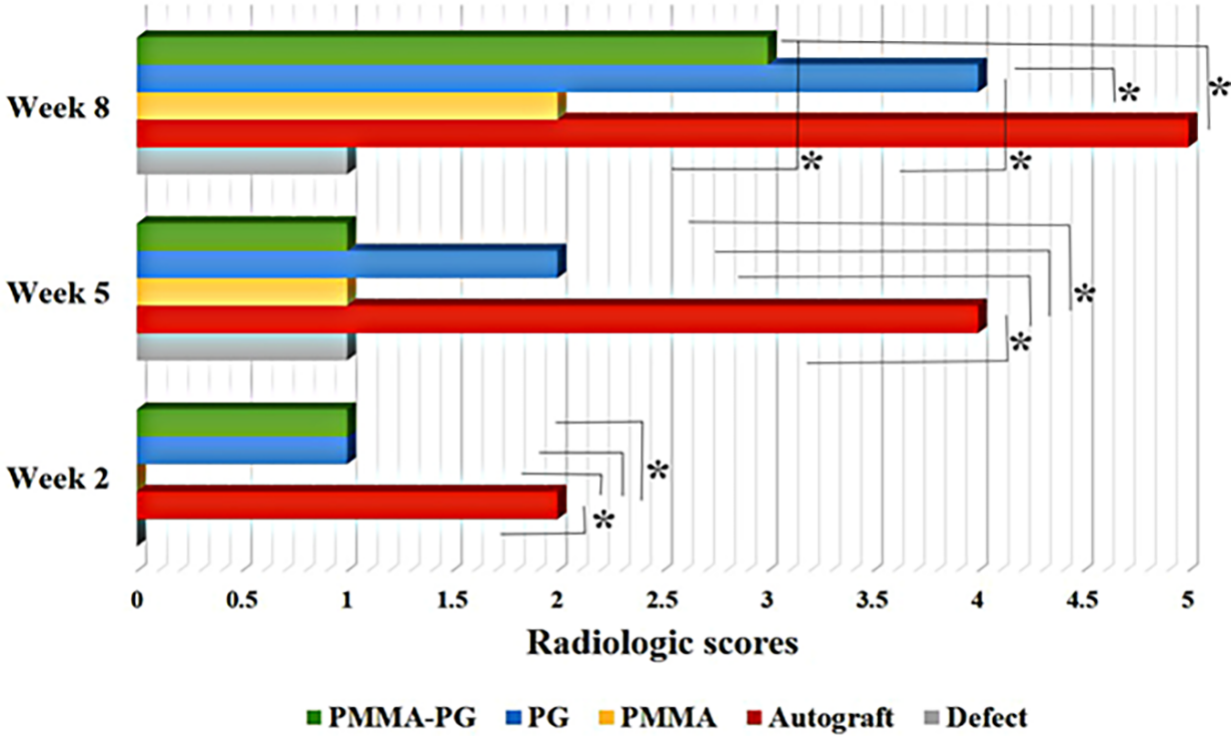
Radiographical findings related to the healing bone defects at various post-operative intervals. Radiological scores in the autograft group were superior to other groups at the 2^nd^ and 5^th^ weeks and to the defect, PMMA and PMMA-PG groups at the 8^th^ week (*P<0*.*05*). Moreover, the significant differences in radiological scores were the PG group with the untreated and PMMA groups and also the PMMA-PG group with the untreated group at the 8^th^ week (*P<0*.*05*). * shows significant differences with *P < 0*.*05*.

The results obtained from radiology at the 2^nd^, 5^th^ and 8^th^ weeks after bone injury have been presented in Figs 2 and 3. The radiographs showed a 75–100% bone formation in the autograft and PG groups, while it was in the range between 25 to 50% for the PMMA-PG group. Both the defect and PMMA groups had the least bone formation (0–25%). Nonetheless, complete remodeling was seen in none of the groups. Bone healing and regeneration in the autograft group was significantly superior to other groups at the 2^nd^ and 5^th^ weeks (*P<0*.*05*). Additionally, the autograft group had significantly higher radiological scores compared with the defect, PMMA and PMMA-PG groups (*P<0*.*05*) at the 8^th^ week after bone injury. Moreover, the defects treated with PG showed significantly greater radiographic scores when compared to the untreated and the PMMA treated bone defects at the 8^th^ week (*P = 0*.*011* and *0*.*046*, respectively). Furthermore, the radiographic scores related to the bone defects in the PMMA-PG groups were significantly higher than those of the untreated group at the 8^th^ week (*P = 0*.*025*).

**Fig 4:**
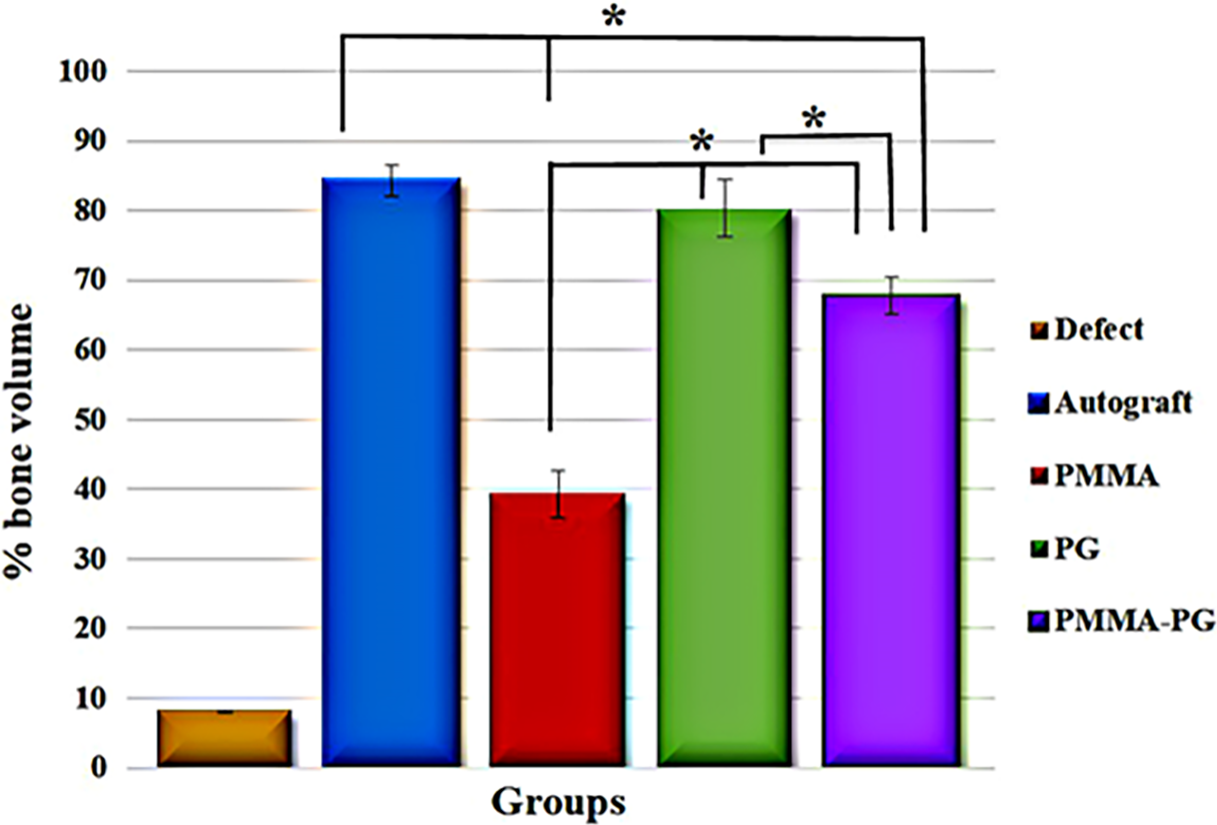
Bone volume (%) of the healed radial bone defects presented as Mean ± SD after eight weeks of injury. All treatment groups had significantly greater bone volume than the defect and PMMA groups (*P<0*.*05*). Amount of bone volume in the autograft and PG groups was superior to other groups (*P<0*.*05*). The bone volume (%) with the PMMA-PG scaffolds was higher than that with the PMMA scaffolds (*P = 0*.*016*). * shows significant differences with *P < 0*.*05*.

The percentages of bone volume in all the treated groups were significantly superior to the untreated (defect) group (*P<0*.*05*). The bone defects treated with the autograft and PG had the highest bone volumes among other groups (*P<0*.*05*). Furthermore, the bone volume (%) related to the defects treated with the PMMA-PG scaffolds was significantly higher as compared to those treated with the PMMA scaffolds (*P = 0*.*016*). The amount of bone volume in the PG group was comparable with that in the autograft group (*P = 0*.*058*) (Figs 2 and 4).

### Histopathologic and histomorphometric findings

**Fig 5:**
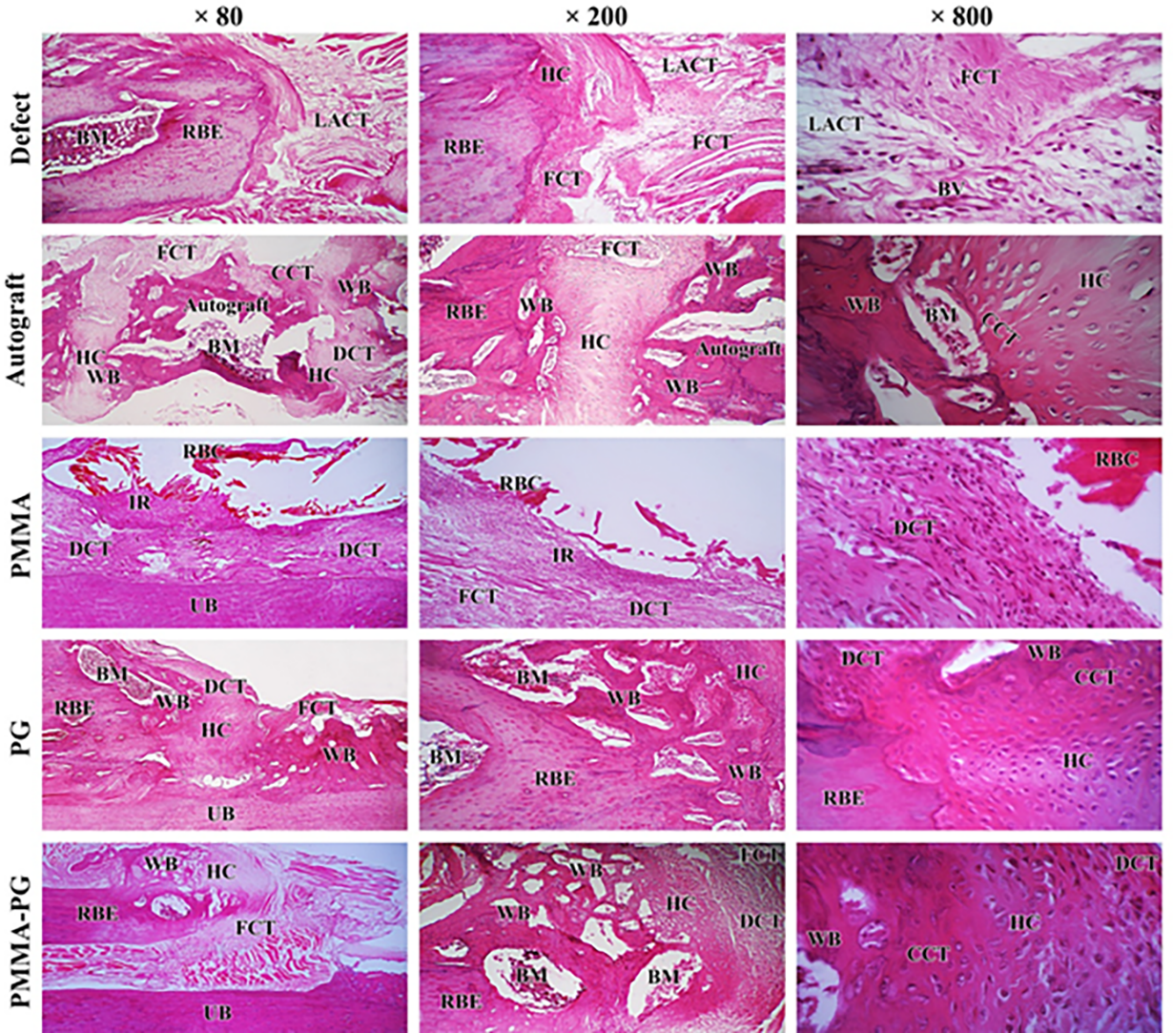
Histopathological view of the longitudinal sections of the rat critical sized radial bone defect at the 8^th^ week post-injury. No remarkable healing has occurred in the untreated defects. The lesions in the defect group are filled with loose areolar or fibrous connective tissue and very few small cartilaginous foci. A non-homogenous matrix composed of fibrous connective tissue, hyaline cartilage and woven bone with bone marrow has filled the defects in the autograft group and the implanted autograft is still seen in the defect area. The PMMA bone cement has not been degraded and it has been surrounded by a large number of mononuclear inflammatory cells and fibrous connective tissue. There is an empty space surrounded by fibrous connective tissue reflecting the PMMA remnants that have been lost during slide preparation procedures. The newly formed woven bone containing bone marrows has filled the edge and the middle part of the defect in the PG treated lesions. Hyaline cartilage and fibrocartilage tissues have connected these two parts of the defect site. The defects in the PMMA-PG treated defects have been filled with a non-homogenous matrix consists of woven bone with bone marrow and hyaline cartilage particularly in the edges, and fibrous connective tissue mostly in the middle region of the defect. Stained with *H&E*. Abbreviations: LACT: Loose areolar connective tissue; FCT: Fibrous connective tissue; RBE: Radial bone edge; WB: Woven bone; BV: Blood vessel; CCT: Calcified cartilaginous tissue; HC: Hyaline cartilage; DCT: Dense connective tissue; BM: Bone marrow; RBC: Remnants of bone cement; IR: Inflammatory reaction; UB: Ulnar bone.

After eight weeks, the injured area in the defect group was replaced with a loose connective tissue containing a large number of fibrocytes + fibroblasts, low density collagen fibers, and numerous blood vessels with few chondrocytes. In fact, the healing process in the untreated defects was still in fibroplasia or proliferation phase and no sign of bone formation and remodeling was found (Fig 5). At the same stage, a non-homogeneous tissue composed of a mixture of fibrocartilage and hyaline cartilage with osseous tissue was observed in the defects treated with autograft. The PG scaffolds were completely degraded and no scaffold remnants were present. New bone formation was particularly remarkable at both proximal and distal ends of the old radial bones in the PG group, which were connected to the middle part of the defect area by fibrocartilage and/or hyaline cartilage tissues.

Some remnants of the implant still existed in the lesions in the PMMA treated defects and did not degrade after eight weeks. These remnants were surrounded by mononuclear inflammatory cells including lymphocytes, plasma cells, macrophages and giant cells and by a fibrous capsule. In addition, few cartilage cells and negligible numbers of osteoblasts were visible in the defects of this group. The PMMA-PG implants were mostly degraded and both edges of the old radial bone regenerated into newly formed woven bone and hyaline cartilage, while fibrocartilage and fibrous connective tissues were present in the middle part of the defect (Fig 5).

The quantitative results relative to microscopic scores and histomorphometric examination of the bone defects after eight weeks of bone injury are available in Tables 2 and 3, respectively. The microscopic scores were given to each group on the basis of the newly formed tissue that filled the defect sites including FCT, hyaline cartilage and bone. Accordingly, when compared to the PMMA and defect groups, the defects treated with the PG, PMMA-PG and autograft had significantly higher microscopic scores and more developed bone healing (*P<0*.*05*).

**Table 3:** Histomorphometric characteristics of healed tissue in the bone defects.

Value (number)	Defect (1)	Autograft (2)	PMMA (3)	PG (4)	PMMA-PG (5)							
	Mean ± SD	Mean ± SD	Mean ± SD	Mean ± SD	Mean ± SD	P^a^	1 vs. 3	1 vs. 4	1 vs. 5	3 vs. 4	3 vs. 5	4 vs. 5
Fibroblast + fibrocyte (n)	193.60 ± 15.52	53.00 ± 6.82^b^	117.40 ± 7.02	59.40 ± 8.20	98.00 ± 10.21	*0*.*000*	*0*.*009*	*0*.*007*	*0*.*009*	*0*.*009*	*0*.*025*	*0*.*018*
Chondroblast + chondrocyte	5.40 ± 1.14	131.40 ± 12.12^c^	19.00 ± 6.53	103.00 ± 6.16	78. 00 ± 7.79	*0*.*000*	*0*.*033*	*0*.*007*	*0*.*009*	*0*.*008*	*0*.*009*	*0*.*014*
Osteoblast + osteocyte	0.00	72.00 ± 11.07^d^	3.00 ± 0.71	86.80 ± 7.36	45.00 ± 6.24	*0*.*000*	*0*.*942*	*0*.*000*	*0*.*000*	*0*.*007*	*0*.*009*	*0*.*011*
Osteoclast	0.00	1.60 ± 0.55^e^	0.00	1.80 ± 0.83	1.00 ± 0.55	*0*.*001*	*1*	*0*.*005*	*0*.*369*	*0*.*005*	*0*.*369*	*0*.*056*
Inflammatory cells	5.60 ± 2.41	21.80 ± 4.15^f^	59.00 ± 12.50	23.40 ± 6.43	24.00 ± 4.69	*0*.*001*	*0*.*009*	*0*.*012*	*0*.*018*	*0*.*016*	*0*.*018*	*0*.*202*
Blood vessels	16.40 ± 2.41	4.60 ± 2.41^g^	11.00 ± 4.60	9.80 ± 1.92	12.00 ± 4.04	*0*.*003*	*0*.*079*	*0*.*093*	*0*.*195*	*0*.*753*	*0*.*994*	*0*.*496*
Osteon	0.00	6.40 ± 2.07^h^	0.00	8.80 ± 1.92	3.20 ± 1.30	*0*.*000*	*1*	*0*.*000*	*0*.*009*	*0*.*000*	*0*.*009*	*0*.*012*
Density of FCT (%)	97.35 ± 0.41	20.48 ± 1.31^i^	83.31 ± 4.05	23.31 ± 2.53	44.77 ± 2.93	*0*.*000*	*0*.*039*	*0*.*009*	*0014*	*0*.*011*	*0*.*025*	*0*.*043*
Density of CT	2.65 ± 0.41	51.00 ± 4.36^j^	14.58 ± 3.74	41.27 ± 2.35	35.03 ± 2.90	*0*.*000*	*0*.*032*	*0*.*008*	*0*.*009*	*0*.*018*	*0*.*034*	*0*.*065*
Density of OT	0.00	28.52 ± 3.67^k^	2.11 ± 0.35	35.52 ± 1.78	20.20 ± 2.45	*0*.*000*	*0*.*537*	*0*.*000*	*0*.*000*	*0*.*009*	*0*.*016*	*0*.*031*

PG: Platelet gel; PMMA: Polymethylmethacrylate; SD: Standard deviation

^a^ One way ANOVA followed by Tukey post-hoc test

^b^ *P<0*.*05* (2 vs. 1, 3, and 5)

^c^ *P<0*.*05* (2 vs. 1, 3, 4, and 5)

^d^ *P<0*.*05* (2 vs. 1, 3, 4, and 5)

^e^ *P = 0*.*000* (2 vs. 1 and 3)

^f^ *P = 0*.*018 and 0*.*014* (2 vs. 1 and 3)

^g^ *P<0*.*05* (2 vs. 1, 3, and 5)

^h^ *P = 0*.*000* (2 vs. 1 and 3)

^i^ *P<0*.*05* (2 vs. 1, 3, and 5)

^j^ *P<0*.*05* (2 vs. 1, 3, 4, and 5)

^k^ *P<0*.*05* (2 vs. 1, 3, 4, and 5)

In terms of histomorphometric examination of the healed bone defects, the highest density of FCT and number of fibrocytes + fibroblasts and the least density of CT and OT and numbers of osteocytes + osteoblasts, chondrocytes + chondroblasts and osteons belonged to the defect group as compared with the treated groups (*P<0*.*05*). At this stage of bone healing, the defects treated with PG had significantly lower density of FCT and number of fibroblasts + fibrocytes, but higher number of chondroblasts + chondrocytes, osteoblasts + osteocyts, osteons, and osteoclasts in comparison to the PMMA and PMMA-PG groups (*P < 0*.*05*). Incorporation of PG into the PMMA implants resulted in significantly reduced numbers of fibroblasts + fibrocytes and density of FCT, and elevated chondrocytes + chondrocytes, osteocytes + osteoblasts, osteons and osteoclasts number and density of CT and OT compared with the PMMA alone (*P<0*.*05*). As the PMMA implants were not degraded and PMMA remnants were still present, the lesions in this group were heavily infiltrated by mononuclear inflammatory cells including macrophages, lymphocytes and plasma cells and multinucleated giant cells (*P<0*.*05*). The PG treated defects had significantly lower number of chondroblasts + chondrocytes and density of CT, but greater number of osteoblasts + osteocytes and osteons and density of OT as compared with the autograft group (*P < 0*.*05*).

The density of newly formed tissues in the defect areas of all groups have been brought in Table 3. In fact, FCT was the main constituent in the untreated defect and PMMA groups, whereas cartilage and bone tissues formed the predominant tissues in the autograft and PG followed by the PMMA-PG groups.

### Scanning electron microscopic analysis

**Fig 6:**
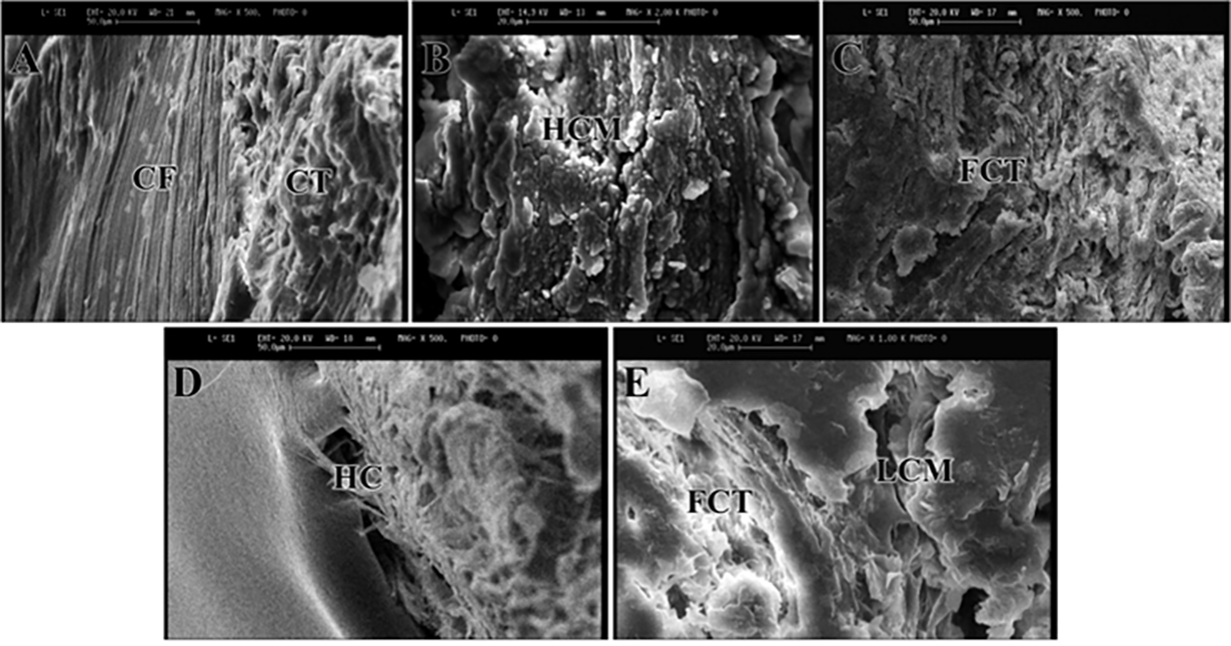
Scanning ultra-micrographs of the rat healed radial bone defects at 8^th^ week post-injury. Collagen fibrils in fibrous connective tissue have filled the gap in the defect group (A), while the defects treated with autograft are filled with calcified bone matrix, cartilaginous tissue and hydroxyapatite crystals (B). The defects in the PMMA group are filled with dense connective tissue and fibrocartilage matrix (C). Calcified bone matrix with a Haversian canal are seen in the PG treated defects (D). The defects in the PMMA-PG group are filled with low calcified bone matrix and fibrocartilage tissue cartilage (E). Abbreviations: CF: Collagen fibrils; CT: Connective tissue; HCM: Highly calcified matrix; FCT: Fibro-cartilaginous tissue; HC: Haversian canal; LCM: Low calcified matrix.

After eight weeks, a loose areolar connective tissue with collagen fibers and fibrils filled the defect area in the untreated group and there was no evidence of HA crystals (Fig 6). A hard callus with calcified hyaline cartilage was observed in the injured area of the autograft group. In addition, the HA crystals were accumulated in dense and loose forms reflecting the non-resorbed parts of the graft and the newly formed bone, respectively. The defect sites in the PMMA treated defects were filled with irregular dense fibrous connective tissue and small amounts of cartilaginous tissue. Calcified hyaline cartilage and hard callus with numerous HA crystals and the Haversian canals were seen in the defected areas of the PG treated group. The defect areas in the PMMA-PG treated group were filled with fibrocartilage tissue and few islets of calcified cartilage and HA crystals. Accumulation of the crystals in the PMMA-PG group was superior to the PMMA group, but inferior to the PG and autograft groups.

### Biomechanical findings

The data obtained from biomechanical examination are available in Table 4. The defect areas of the autograft group showed significantly greater maximum load and stress, but lower stress in comparison to the defect and PMMA groups (*P<0*.*05*). In addition, the PG and PMMA-PG treated defects had significantly higher maximum load (*P = 0*.*011* and *0*.*047*, respectively) and stress (*P = 0*.*014* and *0*.*049*, respectively) compared with the untreated defects. The bone defects in the PG group had significantly lower strain when compared to those in the untreated group (*P = 0*.*016*). All treatment groups showed significantly higher stiffness than the untreated group (*P<0*.*05*). The stiffness of the regenerated bones in the PG and autograft groups was significantly greater in comparison to the defects treated with the PMMA and PMMA-PG scaffolds (*P<0*.*05*). The PG group had significantly lower stiffness compared with the autograft group (*P = 0*.*043)*.

**Table 4:** Biomechanical performance of the injured treated and untreated bones on the 8^th^ post-operative week.

Three point bending test criteria	Defect (1)	Autograft (2)	PMMA (3)	PG (4)	PMMA-PG (5)							
	Mean ± SD	Mean ± SD	Mean ± SD	Mean ± SD	Mean ± SD	P^a^	1 vs. 3	1 vs. 4	1 vs. 5	3 vs. 4	3 vs. 5	4 vs. 5
Maximum load (N)	20.00 ± 3.53	34.80 ± 4.80^b^	25.00 ± 3.54	31.20 ± 4.10	27.60 ± 3.71	*0*.*003*	*0*.*308*	*0*.*011*	*0*.*047*	*0*.*139*	*0*.*837*	*0*.*616*
Stress (N/mm^2^)	2.83 ± 0.50	4.93 ± 0.68^c^	3.54 ± 0.50	4.42 ± 0.59	3.89 ± 0.50	*0*.*003*	*0*.*300*	*0*.*014*	*0*.*049*	*0*.*135*	*0*.*853*	*0*.*584*
Strain (%)	4.99 ± 0.49	3.69 ± 0.26^d^	4.53 ± 0.41	3.81 ± 0.26	4.14 ± 0.53	*0*.*004*	*0*.*482*	*0*.*016*	*0*.*107*	*0*.*067*	*0*.*874*	*0*.*350*
Stiffness (N/mm)	24.88 ± 2.52	57.82 ± 3.83^e^	34.60 ± 3.98	50.96 ± 2.61	40.1 ± 3.60	*0*.*000*	*0*.*032*	*0*.*010*	*0*.*008*	*0*.*018*	*0*.*088*	*0*.*039*

PG: Platelet gel; PMMA: Polymethylmethacrylate; SD: Standard deviation

^a^ Kruskal-Wallis non-parametric ANOVA

^b^ *P<0*.*05* (2 vs. 1 and 3)

^c^ *P<0*.*05* (2 vs. 1 and 3)

^d^ *P<0*.*05* (2 vs. 1 and 3)

^e^ *P<0*.*05* (2 vs. 1, 3, 4, and 5) by Mann-Whitney U test

## Discussion

In this study, we fabricated a new implant composed of human PG and PMMA bone cement to enhance bone healing of radial defects in rats. Given the considerable amount of bone volume in computed scanning and bone formation at the two edges and also the center of defect site indicating continuous bone formation in histopathology and histomorphometric findings of the PG group, we can claim that PG is osteoinductive and osteoconductive and thus it can be used in regeneration of bone defects in the field of BTE. More importantly, incorporation of PG into PMMA cement could improve the healing of bone defects compared with PMMA alone. Consistent with considerable PMMA remnants in gross morphology and histopathology, severe inflammatory reaction including mononuclear cells and multinuclear giant cells, and capsules of fibrous connective tissue around the remnants were found in histology and histopathology. It can be elicited that PMMA alone has low biodegradability, biocompatibility, and bioactivity *in vivo*, while its capacity to regenerate new bone could be improved by the addition of PG. In accordance with our findings, Wildemann et al. [33] indicated that no indication of a foreign body reaction due to the use of biomaterials or growth factors indicate their good biocompatibility and safety.

Although PG alone was more efficient than when it was incorporated into PMMA and bone formation and amount of bone volume was more remarkable, but it resulted in improved healing potential and biological properties of pure PMMA bone cement. In fact, the effectiveness of PMMA-PG was the intermediate of PMMA and PG, so that it was more effective than PMMA, but not as effective as PG. It has previously been demonstrated that PG has great biological properties and on the other hand, growth factors found in platelets can promote the proliferation and differentiation of osteoprogenitor cells and osteoblasts that are possibly responsible for increased new bone formation [19, 24, 26]. The therapeutic outcomes of autologous PRP remain widely controversial, so that a number of studies have reported no positive or even negative effects of this therapeutic modality on bone regeneration [17, 21]. For instance, van Bergen and coworkers [22] indicated that PRP could not enhance the regenerative capacity of demineralized bone matrix in the treatment of osteochondral defects of the talus in goats.

This controversy has been contributed to some variables such as platelet number and preparation procedures [16, 24]. Alternatively, PRP from allogeneic or xenogeneic sources could be used to avoid the additional procedures to harvest large quantity of blood from patients [24]. However, administration of these types of PRP in the BTE applications has rarely been investigated so far, and in particular their immunogenicity in such conditions remains unknown. Zhang et al. [16] revealed that allogeneic PRP possesses negligible immunogenicity and great effectiveness in treatment of critical-sized bilateral radial defects in rabbits. They found a synergetic effect between the allogeneic PRP and the autologous mesenchymal stem cells to promote bone regeneration and this method was considered as a prologue for the development of a new therapeutic strategy in treating large bone defects.

Some other studies used allogeneic PRP in combination with different materials and mesenchymal cells and revealed dramatic effects of PRP on healing and regeneration of calvarial and long bone defects [34, 35]. In addition to our study in a rat model, several studies have used xenogeneic human PRP in healing of radial bone defects in rabbits and obtained promising positive results [15, 30]. Niemeyer et al. [36] showed that addition of xenogenous human leukocyte-depleted PRP did not exhibit any immunogenicity and could compensate inferior osteogenic potential of adipose-tissue derived stem cells compared with bone marrow derived mesenchymal stem cells in treating critical sized tibial defects of sheep. Regarding the platelet concentration, Weibrich et al. [37] stated that the beneficial biological effects of PRP appear with an intermediate platelet concentration of 530–1729 × 10^3^ platelet/μl PRP and the amounts below this range (164–373 × 10^3^ platelet/μl) are suboptimal and those beyond it (1845–3200 × 10^3^ platelet/μl) may be associated with a paradoxically inhibitory effect. In our study, the baseline value of platelets in the whole blood was 259.4 ± 41.6 × 10^3^/μl, while the platelet concentration in PG was 1174.3 ± 261.3 ×10^3^/μl (an approximately 450% increase). Between and within the species, the baseline level of the platelet numbers greatly varies so that this variation may have a non-negligible role in the conflicting results reported in various animal studies performed on PRP [38]. An animal experiment by Plachokova et al. [38] showed that human PRP is more potent than the animal-derived PRP. They found that the human PRP mixed human bone graft or HA/TCP significantly promoted new bone formation after 2 weeks in a rat critical-sized cranial defect model, while rat and goat derived PRP had no regenerative effect.

We could achieve more promising healing with PMMA-PG in comparison to plain PMMA bone cement. The positive results might probably be due to the growth factors present in platelets having osteogenic potentials [19, 24, 26] and also due to porous structure of the PMMA-PG implant (Fig 1) and freeze-drying of the scaffold as it has been shown that porous structures can be obtained by freeze-drying [39]. Improved degradation of PMMA-PG, small residuals, its porous structure providing adequate space for bone growth, diminished inflammatory reaction together with. higher density of newly formed cartilage and bone tissues at the defect sites suggested the improved bone healing of PMMA-PG compared with PMMA alone. Therefore, since the only difference between PMMA-PG and PMMA, that is PG, it can be claimed that the presence of such bioactive materials such as PG with high bioactivity and osteoinductive and osteoconductive properties might exert a synergic effect and be responsible for improvement of bone healing with the PMMA-PG scaffold compared with PMMA alone. Fini et al. [10] could improve osteoblast viability and activity *in vitro* and enhance osteoconduction, new bone formation and bone remodeling *in vivo* by combining alpha-TCP with PMMA. The PMMA/α-TCP implants osteointegrated in the trabecular and cortical bone could accelerate bone mineralization after 12 weeks [10].

In another study conducted by Arabmotlagh et al. [40], the fatigue failure of bone after augmentation with PMMA-nanocrystalline HA composite was retarded compared with plain PMMA in the sheep medial femoral condyles for 3- and 6-month follow-up periods. In other words, the bone-composite specimens had higher fatigue life than the PMMA specimens in both periods [40]. In agreement with our finding, the histological investigation in this study indicated that the plain PMMA was separated from the old bone by fibrous tissue, while tight osteointegration was visible with the composite material. Xing and colleagues showed that incorporation of HA particles into the PMMA nanofibrous scaffolds could enhance the biological function of osteoblasts *in vitro* [41]. In another study by Lye et al. [42], they succeeded to improve biocompatibility and bioactivity of PMMA and to support bone ingrowth by PMMA incorporated with beta-TCP in rabbit bilateral mandibular defects. Nonetheless, there is no study regarding the application of PMMA-PG in the field of bone healing.

In conclusion, we found that PMMA alone possesses low bioactive properties, remains for a long time at the defect sites, stimulates remarkable inflammatory reaction, and fails to enhance significantly bone regeneration compared with the spontaneous capacity of the body. However, the mechanical support provided by PMMA had no significant difference with PMMA-PG or even PG. Therefore, it can be inferred that despite low bioactivity and regenerative properties of PMMA, it may provide an initial mechanical support. Overall, alleviated and modulated inflammatory reaction, improved biological features, biodegradability, and bone regenerative properties of PMMA could be achieved by adding PG. In fact, it can be claimed that PG led to improved features of PMMA in the PMMA-PG group compared with PMMA alone.

Although PG itself was bioactive, osteoinductive, osteoconductive, biocompatible and biodegradable because of its complete degradation, noteworthy bone formation and proper biomechanical strength, the bone regenerative potential and bone volume with PMMA-PG was lower than PG alone. But, considering widespread use of PMMA while its shortcomings, we tended to increase biological and healing properties of PMMA by adding PG. Though PMMA-PG was not as effective as PG alone, more important finding was improvement functions of PMMA. However, the mechanical strength excluding stiffness provided by the PMMA-PG scaffold was comparable with PG. In fact, as has previously been confirmed, it seems that the cytokines and growth factors present in platelets might be responsible for this improvement.

Hence, further investigations may be needed to add other biomaterials into PMMA-PG to enhance mechanical and biological properties to a significantly greater extent compared with PG alone. Although we could improve the regenerative properties of PMMA by adding PG, it should be highlighted that this is the first and thus a preliminary study in this regard, and the obtained findings should be investigated in more details regarding the mechanism of more benefits of the PMMA-PG rather than PMMA and generalized to the clinical applications. However, it is strongly recommended to test bioactivity, biocompatibility and biodegradability of the PMMA-PG scaffold subcutaneously and also at the *in vitro* level. Further studies are needed to answer whether bioactivity of growth factors is maintained in the PMMA-PG, how growth factors are released from the PMMA-PG and what is their mechanism of action in improving healing potential of PMMA. The safety or cytotoxicity of the PMMA-PG scaffold can be tested *in vitro* for example by MTT assay.

## Supporting information

S1 Checklist (DOCX)
