# Supplementary material for: Healing potentials of polymethylmethacrylate bone cement combined with platelet gel in the critical-sized radial bone defect of rats
Source: PLoS One. 2018 Apr 2;13(4):e0194751. doi: 10.1371/journal.pone.0194751 (PMC5880368; doi:10.1371/journal.pone.0194751)
Supplement: S1 Checklist — (DOCX) [file pone.0194751.s001.docx]

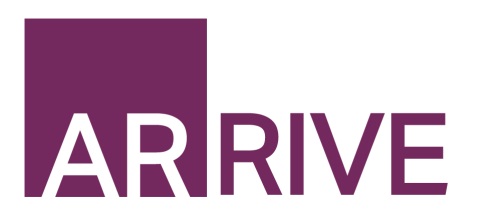


The ARRIVE Guidelines Checklist

Animal Research: Reporting In Vivo Experiments

Ahmad Oryan^1^, Soodeh Alidadi^2^, Amin Bigham-Sadegh^3*^, Ali Moshiri^4^

*^1^ DVM, Ph. D in Comparative pathology, Department of Pathology, School of Veterinary Medicine, Shiraz University, Shiraz, Iran, ^2^DVM, Ph. D Student of Veterinary Pathology, Department of Pathology, School of Veterinary Medicine, Shiraz University, Shiraz, Iran, ^3^DVM, DVSc in Veterinary Surgery, Department of clinical Sciences, School of Veterinary Medicine, Shahrekord University, Shahrekord, Iran, ^4^DVM, DVSc in Veterinary Surgery, Department of Orthopedic Surgery, Faculty of Medicine, AJA University of Medical Science, Tehran, Iran.*

|  | ITEM | RECOMMENDATION | Section/ Paragraph |
| --- | --- | --- | --- |
| Title 1 Healing potentials of polymethylmethacrylate bone cement combined with platelet gel in the critical-sized radial bone defect of rats | | | Title |
| Abstract 2 Polymethylmethacrylate (PMMA) is the most commonly used filler material that lacks biological properties and osteoconductivity or osteoinductivity. Platelet gel (PG) is a typical source of growth factors, cytokines and molecules efficient for bone formation and remodeling. The aim of this study was to evaluate bone healing and regeneration of bone defect in rat model with PMMA with PG. A total of 50 defects were created in the diaphysis of the radii of 25 male Sprague-Dawley rats. These defects were randomly divided into five groups (n = 10 defects for each group) and treated by autograft, plain PMMA, PG and PMMA-PG or left untreated. The rats were examined clinically and radiologically during the experiment and also after euthanasia at the 8th post-operative week, the healed defects were evaluated by gross morphology, histopathology, histomorphometry, computed tomography, scanning electron microscopy and biomechanical testing. The healing in the defects treated with autograft, PG and PMMA-PG was more promising than the untreated and PMMA treated defects. Moreover, bone healing in the defect areas of the PG and PMMA-PG groups was comparable with the autograft group. In conclusion, limited bone regenerative potentials and biological properties of PMMA bone cement such as biodegradability were improved by adding PG in vivo. Therefore, the PG-PMMA can be proposed as a promising option to be applied in bone tissue engineering applications. | | | Abstract |
| INTRODUCTION | | |  |
| Background 3 Among many potential bone cement materials, acrylic bone cement or polymethylmethacrylate (PMMA) has been used in the orthopedic procedures (Hautamaki et al. 2008; Kim et al. 2004). This polymer has been proposed to be suitable in fixation of prosthetic implants and repair of vertebral fractures or vertebroplasty (Arora et al. 2013; Hatten and Voor 2012; Magnan et al. 2013; Wolf-Brandstetter et al. 2013). Other applications of PMMA in the orthopedic field include arthroplasty, remodeling of osteoporotic bones, hip endoprosthesis, hip replacement, and cranioplasty.  Platelet-rich plasma (PRP) which is biodegradable, biocompatible and bioactive and has osteoinductive and osteoconductive capacities can be considered as an appropriate candidate to be added into PMMA (Meimandi-Parizi et al. 2012; Zhang et al. 2013).  PRP can be used in different forms such as injective liquid, gel, sponge and hydrogels (Oryan et al. 2016; Rodriguez et al. 2014). It can be activated to form a gel by addition of calcium chloride or thrombin alone or in combination (Betoni-Junior et al. 2013). This platelet gel (PG) may be applied alone or in combination with different components to provide bone regenerative substitutes (Betoni-Junior et al. 2013; Ogundipe et al. 2011). | | | Paragraph 1  Paragraph 3  Paragraph 4 |
| Objective 4 The healing potential of PMMA with or without xenogenous platelet gel was evaluated in a rat critical-sized radial defect model. | | | Paragraph 4 |
| METHODS | | |  |
| Ethical Statement 5 Human care for all animals was provided in accordance with the Guide for Care and Use of Laboratory Animals published by the National Institutes of Health (NIH publication No. 85-23, revised 1985). The present study was approved by the local Ethics Committee of “Regulations for using animals in scientific procedures” in School of Veterinary Medicine, Shiraz University, Shiraz, Iran. | | | Paragraph 4 |
| Study Design 6 Xenogenous human derived PRP was provided from the Shiraz Blood Bank Center. The two-component PMMA bone cement used in this experiment. The bone defects was created (n = 50, 10 defects in each group) and were either treated with autograft, PMMA, PG alone, PMMA-PG or left empty (defect or untreated group). It should be mentioned that the bone segments harvested from the radii in the defect/untreated group were used as the autologous bone grafs for the autograft group in the same rat. | | | Paragraph 2 |
| Experimental procedure 7 After inserting the implants in the defect areas, the muscles, subcutaneous fascia and skin were sutured in a routine fashion. Post-operative analgesia and antibiotic therapy were provided by intramuscular administration of flunixin meglumine (Razak Co., Tehran, Iran; 2.5 mg/kg) and enrofloxacin (Enrofan 5%, Erfan, Tehran, Iran), respectively for 5 days. | | |  |
| Experimental animals 8 Twenty-five mature male Spraue-Dawley rats weighing 250 ± 25 g were used. | | |  |

The ARRIVE guidelines. Originally published in PLoS Biology, June 20101

| Housing and husbandry 9 Animals were kept in standard cage, in a well-ventilated room with a regular light cycle and with temperature of 21 °C. They were allowed to access to water and food ad libitum. |  | |
| --- | --- | --- |
| Sample size 10 Twenty-five mature male Spraue-Dawley rats weighing 250 ± 25 g were purchased from the Razi Institute, Karaj, Iran. The animals had full access to standard food and water ad libitum throughout the study. General anesthesia was administered by intramuscular injection of Ketamine hydrochloride (Ketamine 10%, 50 mg/kg), Xylazine (Xylazine 2%, 2 mg/kg), and Acepromazine maleate (1mg/kg; all from Alfasa Co., Woerden, Holland). Under aseptic condition, a 2-cm incision was bilaterally made over the forearm and the radii of each animal were exposed. Using an electrical bone saw (Strong. Co. Seoul, South Korea), five mm of the diaphysis of each radius was cut under saline dripping. The created bone defects (n = 50, 10 defects in each group). |  | |
| Allocating animals  to experimental groups 11 Animal grouping was done randomly and all evaluation were done blindly |  | |
| Experimental outcomes 12 Clinical examination, Radiological evaluation, Gross evaluation Three-dimensional computed tomography (3D-CT), Histopathologic and histomorphometric evaluations, Scanning electron microscopy, Biomechanical evaluation, Statistical analysis. |  | |
| Statistical methods 13 The data achieved from the histomorphometric examination were expressed as mean ± SD and analyzed by one-way ANOVA with subsequent Tukey post-hoc tests. The scored values and biomechanical data were statistically compared by Kruskal-Wallis H, non-parametric ANOVA test, and when they were significant, by Mann-Whitney U test. A p-value less than 0.05 was considered to be statistically significant. Statistical analyses were performed by SPSS software, version 16.0 (SPSS, Inc, Chicago, USA). |  | |
| RESULTS |  | |
| Baseline data 14 Animal were health and all vital signs were in normal ranges |  | |
| Numbers analysed 15 500 samples (defected bones) were analysed |  | |
| Outcomes and estimation 16 3.1. Morphology of scaffolds  The SEM micrographs of the PMMA bone cement and PMMA-PG scaffold showed that the pure PMMA was less rough and porous than the PMMA-PG scaffold, so that platelets were observed in some parts of the scaffold (Figure 1).  3.2. Clinical manifestations  All the animals had good appetite and physical activity during the experiment duration and there was no death. Edema, hyperemia, swelling and pain were detectable at the surgery region, in all groups at the first two weeks after the operation. These signs rapidly reduced toward the normal status in the defects treated with PG, PMMA-PG and autograft, so that they appeared almost normal at the 3rd post-operative week. After eight weeks, the untreated defects were empty under digital palpation. In the PG and autograft groups, the defects were filled with a new firm tissue so that the defect site was not detectable. The defects in the PMMA-PG group were filled with a new tissue which was not as firm as the tissue in the PG and autograft groups. The defects treated with PMMA seemed to be filled with a new soft tissue. Regardless of the above descriptions, all animals in the experiment had weight bearing because of the supportive role of the ulna.  3.3. Gross morphology  After eight weeks, the untreated defects were replaced with a soft tissue similar to fascia and the defects were almost empty (Figure 2). The defects treated with PG and autograft were filled with firm tissues and bone union seemed almost complete. In the PMMA group, the implant was not still completely degraded and the defect sites were mostly replaced by fibrous tissue along with the cement remnants. Eventually, firm tissues possibly cartilage or bone filled the defect sites in the PMMA-PG group, but bone union was still incomplete. In addition, very small segments of the PMMA-PG implant were still visible in the defect sites (Figure 2). The bone defects of the autograft and PG groups had significantly higher macroscopic scores compared with those of the defect and PMMA groups (P<0.05) (Table 2). The macroscopic scores related to the defects treated with PMMA-PG were significantly greater than those of the untreated defects (P=0.031). Incorporation of PG into PMMA did not lead to significant difference in macroscopic scores between the PMMA and PMMA-PG groups (P=0.249).  3.4. Diagnostic imaging findings  The results obtained from radiology at the 2nd, 5th and 8th weeks after bone injury have been presented in Figures 2 and 3. The radiographs showed a 75-100% bone formation in the autograft and PG groups, while it was in the range between 25 to 50% for the PMMA-PG group. Both the defect and PMMA groups had the least bone formation (0-25%). Nonetheless, complete remodeling was seen in none of the groups. Bone healing and regeneration in the autograft group was significantly superior to other groups at the 2nd and 5th weeks (P<0.05). Additionally, the autograft group had significantly higher radiological scores compared with the defect, PMMA and PMMA-PG groups (P˂0.05) at the 8th week after bone injury. Moreover, the defects treated with PG showed significantly greater radiographic scores when compared to the untreated and the PMMA treated bone defects at the 8th week (P=0.011 and 0.046, respectively). Furthermore, the radiographic scores related to the bone defects in the PMMA-PG groups were significantly higher than those of the untreated group at the 8th week (P=0.025).  The percentages of bone volume in all the treated groups were significantly superior to the untreated (defect) group (P<0.05). The bone defects treated with the autograft and PG had the highest bone volumes among other groups (P<0.05). Furthermore, the bone volume (%) related to the defects treated with the PMMA-PG scaffolds was significantly higher as compared to those treated with the PMMA scaffolds (P=0.016). The amount of bone volume in the PG group was comparable with that in the autograft group (P=0.058) (Figures 2 and 4).  3.5. Histopathologic and histomorphometric findings  After eight weeks, the injured area in the defect group was replaced with a loose connective tissue containing a large number of fibrocytes + fibroblasts, low density collagen fibers, and numerous blood vessels with few chondrocytes. In fact, the healing process in the untreated defects was still in fibroplasia or proliferation phase and no sign of bone formation and remodeling was found (Figure 5). At the same stage, a non-homogeneous tissue composed of a mixture of fibrocartilage and hyaline cartilage with osseous tissue was observed in the defects treated with autograft.  Some remnants of the implant still existed in the lesions in the PMMA treated defects and did not degrade after eight weeks. These remnants were surrounded by mononuclear inflammatory cells including lymphocytes, plasma cells, macrophages and giant cells and by a fibrous capsule. In addition, few cartilage cells and negligible numbers of osteoblasts were visible in the defects of this group. The PG scaffolds were completely degraded and no scaffold remnants were present. New bone formation was particularly remarkable at both proximal and distal ends of the old radial bones in the PG group, which were connected to the middle part of the defect area by fibrocartilage and/or hyaline cartilage tissues. The PMMA-PG implants were mostly degraded and both edges of the old radial bone regenerated into newly formed woven bone and hyaline cartilage, while fibrocartilage and fibrous connective tissues were present in the middle part of the defect (Figure 5).  The quantitative results relative to microscopic scores and histomorphometric examination of the bone defects after eight weeks of bone injury are available in Tables 2 and 3, respectively. The microscopic scores were given to each group on the basis of the newly formed tissue that filled the defect sites including FCT, hyaline cartilage and bone. Accordingly, when compared to the PMMA and defect groups, the defects treated with the PG, PMMA-PG and autograft had significantly higher microscopic scores and more developed bone healing (P˂0.05).  In terms of histomorphometric examination of the healed bone defects, the highest density of FCT and number of fibrocytes + fibroblasts and the least density of CT and OT and numbers of osteocytes + osteoblasts, chondrocytes + chondroblasts and osteons belonged to the defect group as compared with the treated groups (P˂0.05). At this stage of bone healing, the defects treated with PG had significantly lower density of FCT and number of fibroblasts + fibrocytes, but higher number of chondroblasts + chondrocytes, osteoblasts + osteocyts, osteons, and osteoclasts in comparison to the PMMA and PMMA-PG groups (P ˂ 0.05). Incorporation of PG into the PMMA implants resulted in significantly reduced numbers of fibroblasts + fibrocytes and density of FCT, and elevated chondrocytes + chondrocytes, osteocytes + osteoblasts, osteons and osteoclasts number and density of CT and OT compared with the PMMA alone (P˂0.05). As the PMMA implants were not degraded and PMMA remnants were still present, the lesions in this group were heavily infiltrated by mononuclear inflammatory cells including macrophages, lymphocytes and plasma cells (P˂0.05). The PG treated defects had significantly lower number of chondroblasts + chondrocytes and density of CT, but greater number of osteoblasts + osteocytes and osteons and density of OT as compared with the autograft group (P ˂ 0.05).  The density of newly formed tissues in the defect areas of all groups have been brought in Table 3. In fact, FCT was the main constituent in the untreated defect and PMMA groups, whereas cartilage and bone tissues formed the predominant tissues in the autograft and PG followed by the PMMA-PG groups.  3.6. Scanning electron microscopic analysis  After eight weeks, a loose areolar connective tissue with collagen fibers and fibrils filled the defect area in the untreated group and there was no evidence of HA crystals (Figure 6). A hard callus with calcified hyaline cartilage was observed in the injured area of the autograft group. In addition, the HA crystals were accumulated in dense and loose forms reflecting the non-resorbed parts of the graft and the newly formed bone, respectively. The defect sites in the PMMA treated defects were filled with irregular dense fibrous connective tissue and small amounts of cartilaginous tissue. Calcified hyaline cartilage and hard callus with numerous HA crystals and the Haversian canals were seen in the defected areas of the PG treated group. The defect areas in the PMMA-PG treated group were filled with fibrocartilage tissue and few islets of calcified cartilage and HA crystals. Accumulation of the crystals in the PMMA-PG group was superior to the PMMA group, but inferior to the PG and autograft groups.  3.7. Biomechanical findings  The data obtained from biomechanical examination are available in Table 4. The defect areas of the autograft group showed significantly greater maximum load and stress, but lower stress in comparison to the defect and PMMA groups (P˂0.05). In addition, the PG and PMMA-PG treated defects had significantly higher maximum load (P=0.011 and 0.047, respectively) and stress (P=0.014 and 0.049, respectively) compared with the untreated defects. The bone defects in the PG group had significantly lower strain when compared to those in the untreated group (P=0.016). All treatment groups showed significantly higher stiffness than the untreated group (P˂0.05). The stiffness of the regenerated bones in the PG and autograft groups was significantly greater in comparison to the defects treated with the PMMA and PMMA-PG scaffolds (P˂0.05). The PG group had significantly lower stiffness compared with the autograft group (P=0.043). |  | |
| Advers events 17 We did not observed any advers effects. Hence, further investigations may be needed to add other biomaterials into PMMA-PG to enhance mechanical and biological properties to a significantly greater extent compared with PG alone. Although we could improve the regenerative properties of PMMA by adding PG, it should be highlighted that this is the first and thus a preliminary study in this regard, and the obtained findings should be investigated in more details regarding the mechanism of more benefits of the PMMA-PG rather than PMMA and generalized to the clinical applications. However, it is strongly recommended to test bioactivity, biocompatibility and biodegradability of the PMMA-PG scaffold subcutaneously and also at the in vitro level. Further studies are needed to answer whether the effectiveness of the PMMA-PG in bone healing has correlation with growth factors of PG. The safety or cytotoxicity of the PMMA-PG scaffold can be tested in vitro. |  | |
| DISCUSSION |  | |
| Interpretation/  scientific implications 18 In this study, we fabricated a new implant composed of human PG and PMMA bone cement to enhance bone healing of radial defects in rats. Incorporation of PG into PMMA cement improved the healing of bone defects compared with PMMA alone. Given the considerable amount of bone volume in computed scanning and bone formation at the two edges and also the center of defect site indicating continuous bone formation in histopathology of the PG group, we can claim that PG is osteoinductive and osteoconductive and thus it can be used in regeneration of bone defects in the field of BTE. Due to the presence of PMMA remnants and considerable mononuclear inflammatory reaction and capsules of fibrous connective tissue around them after eight weeks, it can be elicited that PMMA alone has low biodegradability, biocompatibility, and bioactivity in vivo, while its regenerative potential can be improved by the addition of PG. Although PG alone was more efficient than when it was incorporated into PMMA and bone formation and amount of bone volume was more remarkable, but it resulted in improved healing potential and biological properties of pure PMMA bone cement. It has previously been demonstrated that the growth factors found in platelets can promote the proliferation and differentiation of osteoprogenitor cells and osteoblasts that are possibly responsible for increased new bone formation (Oryan et al. 2017; Oryan et al. 2016; Rodriguez et al. 2014).The therapeutic outcomes of autologous PRP remain widely controversial, so that a number of studies have reported no positive or even negative effects of this therapeutic modality on bone regeneration (Khairy et al. 2013; Peng et al. 2016). For instance, van Bergen and coworkers (van Bergen et al. 2013) indicated that PRP could not enhance the regenerative capacity of demineralized bone matrix in the treatment of osteochondral defects of the talus in goats. This controversy has been contributed to some variables such as platelet number and preparation procedures (Oryan et al. 2016; Zhang et al. 2013). Alternatively, PRP from allogeneic or xenogeneic sources could be used to avoid the additional procedures to harvest large quantity of blood from patients (Oryan et al. 2016). However, administration of these types of PRP in the BTE applications has rarely been investigated so far, and in particular their immunogenicity in such conditions remains unknown. Zhang et al. (Zhang et al. 2013) revealed that allogeneic PRP possesses negligible immunogenicity and great effectiveness in treatment of critical-sized bilateral radial defects in rabbits. They found a synergetic effect between the allogeneic PRP and the autologous mesenchymal stem cells to promote bone regeneration and this method was considered as a prologue for the development of a new therapeutic strategy in treating large bone defects.  Some other studies used allogeneic PRP in combination with different materials and mesenchymal cells and revealed dramatic effects of PRP on healing and regeneration of calvarial and long bone defects (He et al. 2015; Tajima et al. 2014). In addition to our study in a rat model, several studies have used xenogeneic human PRP in healing of radial bone defects in rabbits and obtained promising positive results (Meimandi-Parizi et al. 2012; Oryan et al. 2012). Niemeyer et al. (Niemeyer et al. 2010) showed that addition of xenogenous human leukocyte-depleted PRP did not exhibit any immunogenicity and could compensate inferior osteogenic potential of adipose-tissue derived stem cells compared with bone marrow derived mesenchymal stem cells in treating critical sized tibial defects of sheep. Furthermore, several studies applied xenogenous bovine-derived PG embedded within three-dimensional collagen implants in regeneration of the Achilles tendon defect in rabbits and confirmed the effectiveness of PG without any side effects and immunogenicity (Moshiri et al. 2015; Oryan et al. 2014a; Oryan et al. 2014b).  Regarding the platelet concentration, Weibrich et al. (Weibrich et al. 2004) stated that the beneficial biological effects of PRP appear with an intermediate platelet concentration of 530-1729 × 103 platelet/µl PRP and the amounts below this range (164-373 × 103 platelet/µl) are suboptimal and those beyond it (1845-3200 × 103 platelet/µl) may be associated with a paradoxically inhibitory effect. In our study, the baseline value of platelets in the whole blood was 259.4 ± 41.6 × 103/µl, while the platelet concentration in PG was 1174.3 ± 261.3 ×103/µl (an approximately 450 % increase). Between and within the species, the baseline level of the platelet numbers greatly varies so that this variation may have a non-negligible role in the conflicting results reported in various animal studies performed on PRP (Plachokova et al. 2009). An animal experiment by Plachokova et al. (Plachokova et al. 2009)showed that human PRP is more potent than the animal-derived PRP. They found that the human PRP mixed human bone graft or HA/TCP significantly promoted new bone formation after 2 weeks in a rat critical-sized cranial defect model, while rat and goat derived PRP had no regenerative effect.  We could achieve more promising healing with PMMA-PG in comparison to plain PMMA bone cement. The positive results may probably be due to the growth factors present in platelets having osteogenic potentials (Oryan et al. 2017; Oryan et al. 2016; Rodriguez et al. 2014) and also due to porous macro-architecture of the implant by adding PG and creating some pores in the PMMA cement (Figure 1) and freeze-drying of the scaffold as it has been shown that porous structures can be obtained by freeze-drying (Lv and Feng 2006). Therefore, it can be claimed that the presence of such bioactive materials such as PG with high bioactivity and osteoinductive and osteoconductive properties might exert a synergic effect and be responsible for improvement of bone healing with the PMMA-PG scaffold. Fini et al. (Fini et al. 2002) could improve osteoblast viability and activity in vitro and enhance osteoconduction, new bone formation and bone remodeling in vivo by combining alpha-TCP with PMMA. The PMMA/α-TCP implants osteointegrated in the trabecular and cortical bone could accelerate bone mineralization after 12 weeks (Fini et al. 2002)..  In another study conducted by Arabmotlagh et al. (Arabmotlagh et al. 2014), the fatigue failure of bone after augmentation with PMMA-nanocrystalline HA composite was retarded compared with plain PMMA in the sheep medial femoral condyles for 3- and 6-month follow-up periods. In other words, the bone-composite specimens had higher fatigue life than the PMMA specimens in both periods (Arabmotlagh et al. 2014). In agreement with our finding, the histological investigation in this study indicated that the plain PMMA was separated from the old bone by fibrous tissue, while tight osteointegration was visible with the composite material. Xing and colleagues showed that incorporation of HA particles into the PMMA nanofibrous scaffolds could enhance the biological function of osteoblasts in vitro (Xing et al. 2013). In another study by Lye et al. (Lye et al. 2013), they succeeded to improve biocompatibility and bioactivity of PMMA and to support bone ingrowth by PMMA incorporated with beta-TCP in rabbit bilateral mandibular defects. Nonetheless, there is no study regarding the application of PMMA-PG in the field of bone healing. |  | |
| Generalisability translation 19 we found that PMMA alone possesses low bioactive properties, remains for a long time at the defect sites, stimulates remarkable inflammatory reaction, and fails to enhance significantly bone regeneration compared with the spontaneous capacity of the body. However, the mechanical support provided by PMMA had no significant difference with PMMA-PG or even PG. Therefore, it can be inferred that despite low bioactivity and regenerative properties of PMMA, it may provide an initial mechanical support. Overall, alleviated inflammatory reaction, improved biological features, biodegradability, and bone regenerative properties of PMMA could be achieved by adding PG. PG alone was bioactive, osteoinductive, osteoconductive, biocompatible and biodegradable because of its complete degradation, noteworthy bone formation and proper biomechanical strength without any side effect after eight weeks. In fact, as has previously been confirmed, it seems that the cytokines and growth factors present in platelets are responsible for this improvement |  | |
| Funding 20 We have not any funding for doing this research |  |  |

References:
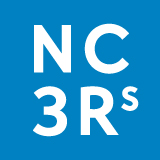


Arabmotlagh M, Bachmaier S, Geiger F, and Rauschmann M. 2014. PMMA-hydroxyapatite composite material retards fatigue failure of augmented bone compared to augmentation with plain PMMA: In vivo study using a sheep model. Journal of Biomedical Materials Research Part B: Applied Biomaterials 102(8):1613-1619.

Arora M, Chan EK, Gupta S, and Diwan AD. 2013. Polymethylmethacrylate bone cements and additives: A review of the literature. World J Orthop 4(2):67-74.

Betoni-Junior W, Dechichi P, Esteves JC, Zanetta-Barbosa D, and Magalhأ£es AEOr. 2013. Evaluation of the bone healing process utilizing platelet-rich plasma activated by thrombin and calcium chloride: a histologic study in rabbit calvaria. Journal of Oral Implantology 39(1):14-21.

Fini M, Giavaresi G, Aldini NN, Torricelli P, Botter R, Beruto D, and Giardino R. 2002. A bone substitute composed of polymethylmethacrylate and alpha-tricalcium phosphate: results in terms of osteoblast function and bone tissue formation. Biomaterials 23(23):4523-4531.

Hatten HP, and Voor MJ. 2012. Bone healing using a bi-phasic ceramic bone substitute demonstrated in human vertebroplasty and with histology in a rabbit cancellous bone defect model. Interventional Neuroradiology 18(1):105-113.

Hautamaki MP, Aho AJ, Alander P, Rekola J, Gunn J, Strandberg N, and Vallittu PK. 2008. Repair of bone segment defects with surface porous fiber-reinforced polymethyl methacrylate (PMMA) composite prosthesis: Histomorphometric incorporation model and characterization by SEM. Acta orthopaedica 79(4):555-564.

He F, Chen Y, Li J, Lin B, Ouyang Y, Yu B, Xia Y, and Ye J. 2015. Improving bone repair of femoral and radial defects in rabbit by incorporating PRP into PLGA/CPC composite scaffold with unidirectional pore structure. Journal of Biomedical Materials Research Part A 103(4):1312-1324.

Khairy NM, Shendy EE, Askar NA, and El-Rouby DH. 2013. Effect of platelet rich plasma on bone regeneration in maxillary sinus augmentation (randomized clinical trial). International journal of oral and maxillofacial surgery 42(2):249-255.

Kim SB, Kim YJ, Yoon TL, Park SA, Cho IH, Kim EJ, Kim IA, and Shin J-W. 2004. The characteristics of a hydroxyapatite-chitosan-PMMA bone cement. Biomaterials 25(26):5715-5723.

Lv Q, and Feng Q. 2006. Preparation of 3-D regenerated fibroin scaffolds with freeze drying method and freeze drying/foaming technique. Journal of Materials Science: Materials in Medicine 17(12):1349-1356.

Lye KW, Tideman H, Wolke JCG, Merkx MAW, Chin FKC, and Jansen JA. 2013. Biocompatibility and bone formation with porous modified PMMA in normal and irradiated mandibular tissue. Clinical oral implants research 24(A100):100-109.

Magnan B, Bondi M, Maluta T, Samaila E, Schirru L, and DallâOca C. 2013. Acrylic bone cement: current concept review. Musculoskeletal surgery 97(2):93-100.

Meimandi-Parizi A, Oryan A, Shafiei-Sarvestani Z, and Bigham AS. 2012. Human platelet rich plasma plus Persian Gulf coral effects on experimental bone healing in rabbit model: radiological, histological, macroscopical and biomechanical evaluation. Journal of Materials Science: Materials in Medicine 23(2):473-483.

Moshiri A, Oryan A, and Meimandi-Parizi A. 2015. Synthesis, development, characterization and effectiveness of bovine pure platelet gel-collagen-polydioxanone bioactive graft on tendon healing. Journal of cellular and molecular medicine 19(6):1308-1332.

Niemeyer P, Fechner K, Milz S, Richter W, Suedkamp NP, Mehlhorn AT, Pearce S, and Kasten P. 2010. Comparison of mesenchymal stem cells from bone marrow and adipose tissue for bone regeneration in a critical size defect of the sheep tibia and the influence of platelet-rich plasma. Biomaterials 31(13):3572-3579.

Ogundipe OK, Ugboko VI, and Owotade FJ. 2011. Can autologous platelet-rich plasma gel enhance healing after surgical extraction of mandibular third molars? Journal of Oral and Maxillofacial Surgery 69(9):2305-2310.

Oryan A, Alidadi S, Bigham-Sadegh A, Moshiri A, and Kamali A. 2017. Effectiveness of tissue engineered chitosan-gelatin composite scaffold loaded with human platelet gel in regeneration of critical sized radial bone defect in rat. Journal of controlled release : official journal of the Controlled Release Society 254:65-74.

Oryan A, Alidadi S, and Moshiri A. 2016. Platelet-rich plasma for bone healing and regeneration. Expert opinion on biological therapy 16:213-232.

Oryan A, Moshiri A, and Meimandi-Parizi A. 2014a. Role of embedded pure xenogenous bovine platelet gel on experimental tendon healing, modelling and remodelling. BioDrugs 28(6):537-556.

Oryan A, Moshiri A, Meimandi-Parizi A, and Maffulli N. 2014b. Role of xenogenous bovine platelet gel embedded within collagen implant on tendon healing: an in vitro and in vivo study. Experimental Biology and Medicine 240:194-210.

Oryan A, Parizi AM, Shafiei-Sarvestani Z, and Bigham AS. 2012. Effects of combined hydroxyapatite and human platelet rich plasma on bone healing in rabbit model: radiological, macroscopical, hidtopathological and biomechanical evaluation. Cell and tissue banking 13(4):639-651.

Peng W, Kim I-k, Cho H-y, Seo J-H, Lee D-H, Jang J-M, and Park S-H. 2016. The healing effect of platelet-rich plasma on xenograft in peri-implant bone defects in rabbits. Maxillofacial plastic and reconstructive surgery 38(1):1.

Plachokova AS, Van den Dolder J, van den Beucken J, and Jansen JA. 2009. Bone regenerative properties of rat, goat and human platelet-rich plasma. International journal of oral and maxillofacial surgery 38(8):861-869.

Rodriguez IA, Growney Kalaf EA, Bowlin GL, and Sell SA. 2014. Platelet-rich plasma in bone regeneration: engineering the delivery for improved clinical efficacy. BioMed research international 2014.

Tajima S, Tobita M, Orbay H, Hyakusoku H, and Mizuno H. 2014. Direct and indirect effects of a combination of adipose-derived stem cells and platelet-rich plasma on bone regeneration. Tissue Engineering Part A 21(5-6):895-905.

van Bergen CJA, Kerkhoffs GMMJ, أ–zdemir M, Korstjens CM, Everts V, van Ruijven LJ, van Dijk CN, and Blankevoort L. 2013. Demineralized bone matrix and platelet-rich plasma do not improve healing of osteochondral defects of the talus: an experimental goat study. Osteoarthritis and Cartilage 21(11):1746-1754.

Weibrich G, Hansen T, Kleis W, Buch R, and Hitzler WE. 2004. Effect of platelet concentration in platelet-rich plasma on peri-implant bone regeneration. Bone 34(4):665-671.

Wolf-Brandstetter C, Roessler S, Storch S, Hempel U, Gbureck U, Nies B, Bierbaum S, and Scharnweber D. 2013. Physicochemical and cell biological characterization of PMMA bone cements modified with additives to increase bioactivity. Journal of Biomedical Materials Research Part B: Applied Biomaterials 101(4):599-609.

Xing Z-C, Han S-J, Shin Y-S, Koo T-H, Moon S, Jeong Y, and Kang I-K. 2013. Enhanced osteoblast responses to poly (methyl methacrylate)/hydroxyapatite electrospun nanocomposites for bone tissue engineering. Journal of Biomaterials Science, Polymer Edition 24(1):61-76.

Zhang Z-Y, Huang A-W, Fan JJ, Wei K, Jin D, Chen B, Li D, Bi L, Wang J, and Pei G. 2013. The potential use of allogeneic platelet-rich plasma for large bone defect treatment: immunogenicity and defect healing efficacy. Cell transplantation 22(1):175-187.
